# Unraveling Acute and Post-COVID Cytokine Patterns to Anticipate Future Challenges

**DOI:** 10.3390/jcm12165224

**Published:** 2023-08-11

**Authors:** Makhabbat Bekbossynova, Ainur Tauekelova, Aliya Sailybayeva, Samat Kozhakhmetov, Karakoz Mussabay, Laura Chulenbayeva, Alibek Kossumov, Zhanagul Khassenbekova, Elizaveta Vinogradova, Almagul Kushugulova

**Affiliations:** 1 National Research Cardiac Surgery Center, Astana 020000, Kazakhstan; astanamaha@gmail.com (M.B.); auekelovaajnura@gmail.com (A.T.); dr.alisai@gmail.com (A.S.); 2 Center for Life Sciences, National Laboratory Astana, Nazarbayev University, Astana 010000, Kazakhstan; skozhakhmetov@nu.edu.kz (S.K.); laura.chulenbayeva@nu.edu.kz (L.C.); alibek.kossumov@nu.edu.kz (A.K.); st.paulmississippi@gmail.com (E.V.); 3 Department of Microbiology and Virology Named after Sh.I.Sarbasova, Astana Medical University, Astana 010000, Kazakhstan; karakoz.musabay@gmail.com; 4 Department of General Pharmacology, Astana Medical University, Astana 010000, Kazakhstan; zhanagul.khassenbekova@gmail.com; 5 Almagul Kushugulova, Laboratory of Microbiome, Center for Life Sciences, National Laboratory Astana, Nazarbayev University, Kabanbay Batyr Ave., 53, Block S1, Office 303, Astana 010000, Kazakhstan

**Keywords:** SARS-CoV-2, post-COVID, cytokines, COVID-19, PASC

## Abstract

The aims of this study were to analyze cytokine profiles in patients with COVID-19, gain insights into the immune response during acute infection, identify cytokines associated with disease severity and post-COVID complications, and explore potential biomarkers for prognosis and therapeutic targets. Using a multiplex analysis, we studied the cytokine pattern in 294 acute COVID-19 and post-COVID patients with varying severities of infection. Our findings revealed that disease severity was associated with elevated levels of IL-15, IL-8, and fractalkine. Severe/extremely severe forms in comparison with mild/moderate disease were associated with MCP-1, IFNa2, IL-7, IL-15, EGF, IP-10, IL-8, Eotaxin, FGF-2, GROa, sCD40L, and IL-10. The key cytokines of post-COVID are FGF-2, VEGF-A, EGF, IL-12(p70), IL-13, and IL-6. By the sixth month after recovering from a coronavirus infection, regardless of disease severity, some patients may develop complications such as arterial hypertension, type 2 diabetes mellitus, glucose intolerance, thyrotoxicosis, atherosclerosis, and rapid progression of previously diagnosed conditions. Each complication is characterized by distinct cytokine profiles. Importantly, these complications can also be predicted during the acute phase of the coronavirus infection. Understanding cytokine patterns can aid in predicting disease progression, identifying high-risk patients, and developing targeted interventions to improve the outcomes of COVID-19.

## 1. Introduction

The COVID-19 pandemic is not yet fully resolved, despite a notable decrease in new SARS-CoV-2 infections. However, we are already grappling with new challenges concerning post-COVID pathologies. These complications can affect individuals who experienced both severe and asymptomatic forms of the infection, presenting a wide array of symptoms and conditions such as respiratory issues, cardiovascular problems, neurological disorders, mental health conditions, fatigue, and cognitive difficulties. Thus, the clinical picture of post-COVID syndrome is polymorphic, and the underlying pathogenetic mechanisms are still unclear.

Factors such as organ and tissue damage, endothelial dysfunction, and autoimmune processes contribute to the complex nature of post-COVID pathologies [1,2,3]. Among the potential mechanisms, ongoing inflammation and immunological disorders, including dysregulation of cytokine responses, are thought to play a significant role [4,5]. 

It is important to recognize that cytokines are not solely involved in immune regulation but also have diverse physiological functions [6,7,8]. Excessive or prolonged production of pro-inflammatory cytokines can lead to chronic inflammation, tissue damage, and autoimmune diseases [9,10,11]. Conversely, inadequate or impaired cytokine production can result in immunodeficiency and heightened vulnerability to infections [12,13].

Certain cytokines are linked to the worsening of COVID-19, triggering an excessive immune response and cytokine storm that damage organs, especially the lungs. Elevated levels of IL-6 and IL-8 are observed in severe cases, contributing to respiratory symptoms and lung inflammation [14,15]. Similarly, increased TNF-alpha and IL-1β levels are associated with a dysregulated immune response and organ damage in severe COVID-19 patients [15,16].

By identifying specific cytokines or cytokine profiles associated with various post-COVID conditions, it is potentially possible to gain insight into how the immune response influences disease severity and complications following COVID-19.

The objectives of this study lie in an analysis of the cytokine profiles of patients with COVID-19, in providing insights into the immune response during acute infection, in identifying cytokines associated with disease severity and post-COVID complications, and in exploring potential biomarkers for prognosis and therapeutic targets. Understanding the cytokine patterns can help in predicting disease progression, identifying high-risk patients, and developing targeted interventions to improve outcomes in COVID-19.

## 2. Materials and Methods

### 2.1. Study Design and Participants

In this cohort study conducted from May 2021 to September 2022 at the National Scientific Cardiac Surgery Center (NSCSC), we included 294 adults aged 18 years or older who had confirmed COVID-19, as well as 40 SARS-CoV-2 negative controls. The study was approved by the Local Ethics Committee (reference number 01-91/2021 dated 22 April 2021) of JSC “National Scientific Cardiac Surgery Center”, and all participants provided written informed consent. All methods were conducted in accordance with the guidelines and regulations of the Kazakhstan Health Care system.

Baseline demographics, clinical characteristics, comorbid conditions, instrumental investigation data, vital signs, and laboratory values were collected by trained hospital personnel and are presented in Table 1. Serum samples for studying immunological parameters were obtained on days 1–3 from the onset of illness (T0) and on day 15 of observation (T1) during the acute phase of the disease. Subsequently, we observed the patients over time, and serum samples were collected at the following intervals: T2—1 month of observation, T3—3 months of observation, T4—6 months of observation, and T5—9 months of observation. 

The severity levels were determined according to the clinical protocol for the diagnosis and treatment of COVID-19 in adults approved by the Ministry of Health of the Republic of Kazakhstan on 28 January 2022 (Protocol No156), which is based on WHO guidelines and recommendations for the clinical management of patients with COVID-19 and its severity. The severity of COVID-19 is determined based on clinical symptoms, inflammatory markers, respiratory function, blood oxygen saturation levels, X-ray findings, underlying disease, age, and laboratory test scores [17,18]. 

### 2.2. Cytokine/Chemokine Analysis

Luminex assays were performed at the laboratories of Center for Life Sciences National Laboratory Astana, Nazarbayev University, using Milliplex HCYTMAG60PMX41BK kits from EMD Millipore Corporation (Bedford, MA, USA). Studies were performed using a BioRad (Hercules, CA, USA) Bio-Plex 200 system. Serum samples were frozen and stored at −80 °C and analyses were performed according to the manufacturer’s recommendations for serum samples, utilizing recommended sample dilutions and standard curve concentrations (Merck-Millipore, Molsheim, France).

### 2.3. Statistics

All statistical calculations were performed in Python 3 using NumPy 1.21.5, SciPy 1.7.0, statsmodels 0.13.5, and scikit-posthocs 0.7.0 libraries. Visualization was performed using matplotlib 3.7.1, seaborn 0.11.2, matplotlib-venn 0.11.9, and sankeyflow 0.3.8 libraries. Principal component analysis (PCA), clustering, and feature importance analysis were performed using scikit-learn 1.2.2. All correlation analyses and rank tests were performed using the Benjamini–Hochberg FDR correction at a significance level ≤ 0.05. Correlation analysis was performed with the Spearmanr coefficient for continuous variables, the Point-Biserial coefficient for mixed binary and continuous variable pairs (on log10 transformed data), and the Phi coefficient for binary variables. All negative correlations are shown in blue, the positive in red. For comparison of 3 or more groups, a Kruskal–Wallis test with a post hoc Dunn test for multiple comparisons with FDR correction was used. For comparison of 2 groups, a Mann–Whitney U test with FDR correction was used. Venn diagrams, Sankey diagrams, and correlation clustergrams were constructed based on significant correlations (*p* ≤ 0.05) after FDR correction. Feature importance analysis was based on the calculation of the mean decrease in impurity (MDI), with a higher MDI implying higher importance. MDIs were obtained during leave-one-out cross-validation using the random forest algorithm with default parameters (number of trees = 100) and the “balanced” weighting of classes option. No prior or inner feature selection was performed during cross-validation. The final most important features were assessed using a permutation test (with 1000 repeats and 5 stratified splits). To evaluate the reliability of the discriminatory power of the identified important features, the area under the receiving curve (AUC) and average precision (AP) metrics were calculated during leave-one-out cross-validation and permutation tests. The AUC summarizes the area under the true positive rate–false positive rate curve, while the AP summarizes the area under the recall–precision curve. The latter metric is considered for evaluating performance in the presence of highly imbalanced data. An AUC > 0.5, an AP > 0.2, and a significance < 0.05 (based on the permutation test) were considered to indicate significant descriptive performance and overall importance of identified features. Cluster analysis was performed based on the most significant features identified in the feature importance analysis on log10-transformed standard scaled data using the PCA algorithm and agglomerative clustering (k = 2) with “ward” linkage. The following analysis of the count distribution was performed using Fisher’s exact test. Odds ratios greater than 1 and *p* ≤ 0.05 were considered significant.

## 3. Results

### 3.1. Demographic and Baseline Characteristics

**Figure 1:**
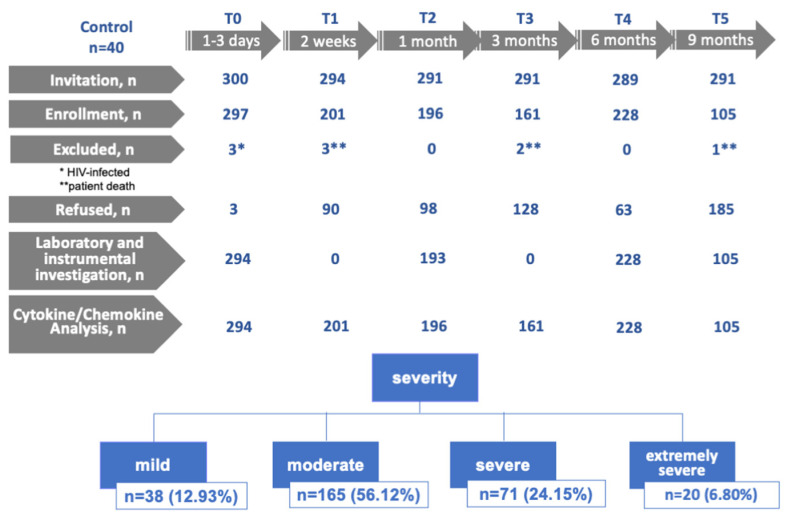
Scheme of the study. Invitation–recruitment of patients; enrollment–patient registration; excluded–registered but subsequently excluded due to unmet condition (* HIV, ** patient death); refused–recruited but subsequently refused to participate; laboratory and instrumental investigation–participated in the laboratory/instrumental tests; cytokine/chemokine analysis–participated in immunologic parameter collection; T0—1–3 days from the onset of illness; T1—15th day of observation (acute phase); T2—1 month of observation; T3—3 months of observation; T4—6 months of observation; T5—9 months of observation.

The study included 294 patients with COVID-19 and 40 in the control group. Out of 294 patients, COVID-19 was confirmed by PCR in 172 patients; the remaining patients were included in the study group symptomatically, but COVID-19 was subsequently confirmed by the study of specific antibodies. Baseline characteristics of COVID-19 and control patients are shown in Table 1. The study scheme is illustrated in Figure 1.

### 3.2. Cytokines Features of Acute COVID-19

During infection with the SARS-CoV-2, the immune system produces various cytokines to help fight the virus. It is worth noting that cytokine levels can vary greatly among people with COVID-19, and not all patients with severe disease experience a cytokine storm. We observed patients in the acute period in 2021, during the predominance of the Delta variant [19], and patients in the acute and post-COVID period (up to 9 months) in 2022, when the Omicron variant predominated [20].

On average patients were characterized by increased levels of pro-inflammatory TNFa (p2021 = 0.034, p2022 = 0.0007), IL-6 (p2021 = 0.14, p2022 = 0.0009), IL-1RA (p2021 = 0.0051, p2022 = 0.73), IL-15 (p2021 < 0.0001, p2022 = 0.1), IL-2 (p2021 = 0.5, p2022 = 0.4), IL-5 (p2021 = 0.51, p2022 < 0.0001), MIP-1b (p2021 = 0.85, p2022 < 0.0001), anti-inflammatory IL-4 (p2021 = 0.25, p2022 = 0.0033), IP-10 (p2021 < 0.0001, p2022 = 0.0051), TGFa (p2021 = 0.0002, p2022 = 0.069), EGF (p2021 < 0.0001, p2022 = 0.45), and VEGF-A (p2021 = 0.0023, p2022 < 0.0001) and reduced values of MIP-1a (p2021 < 0.0001 p2022 = 0.22), IL-8 (p2021 = 0.13, p2022 < 0.0001), IL-9 (p2021 = 0.29, p2022 = 0.74), MCP-1 (p2021 = 0.75, p2022 < 0.0001), Eotaxin (p2021 = 0.33, p2022 < 0.0001), IFNgamma (p2021 = 0.53, p2022 = 0.14). sCD40L (p2021 = 0.4, p2022 < 0.0001), and TNFb (p2021 = 0.82, p2022 = 0.0036) at T0 compared to the control (Table 2). 

**Figure 2:**
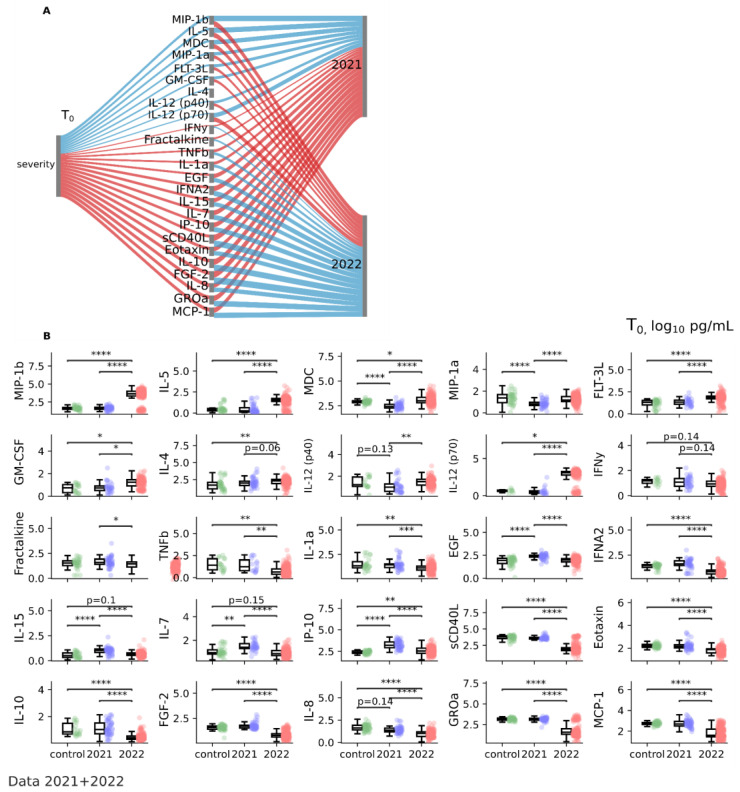
Cytokine profile by year of observation for data collected in 2021 and 2022 at T0 (1–3 days after illness onset). (**A**) Sankey diagram depicting the significant associations between severity, cytokine profile, and year of observation (2021 and 2022). Positive associations are depicted in red and negative associations in blue. Positive association indicates that both variables increase and decrease together. Negative association indicates that while one variable is increasing the other is decreasing and vice versa. The width of the flow indicates the strength of the association (effect size). Spearmanr coefficient for continuous variables and Point-Biserial coefficient for binary-continuous pairs, FDR, *p* < 0.05; (**B**) Boxplots of cytokine levels (log10 pg/mL) by year of observation (2021 and 2022) compared to control with indication of significance. Control data are shown in green, 2021 data in blue and 2022 data in red. Kruskal–Wallis with a *post hoc* Dunn’s test with FDR correction. * *p* < 0.05; ** *p* < 0.01; *** *p* < 0.0001; **** *p* < 0.00001. Data 2021 + 2022 (Supplementary Table S1).

However, despite the apparent similarity, we found a significant difference between cytokine signatures for patients in 2021 and 2022 (Figure 2 and Table 2). When comparing 2021 and 2022 (Figure 2A), in patients in 2022, there was a decrease in the cytokines FGF-2 (*p* < 0.0001), IFNA2 (*p* < 0.0001), IL-1a (*p* < 0.0001), IL-7 (*p* < 0.0001), IL-10 (*p* < 0.0001), and in 2021, IL-1b (*p* = 0.0548), IL-12 (p40) (*p* = 0.0001), MDC (*p* < 0.0001) were reduced. In patients in 2022, we observe increased values; also, in patients in 2022, the values of the cytokines fractalkine (*p* = 0.0023), GM-CSF (*p* < 0.0001), GROa (*p* < 0.0001), and IL-12 (p70) (*p* < 0.0001) were reduced, while we observed norm values among patients in 2021 (Figure 2). Presumably, the differences are associated with the characteristics of the circulating strain and the associated pathogenetic mechanisms of the infectious process.

**Figure 3:**
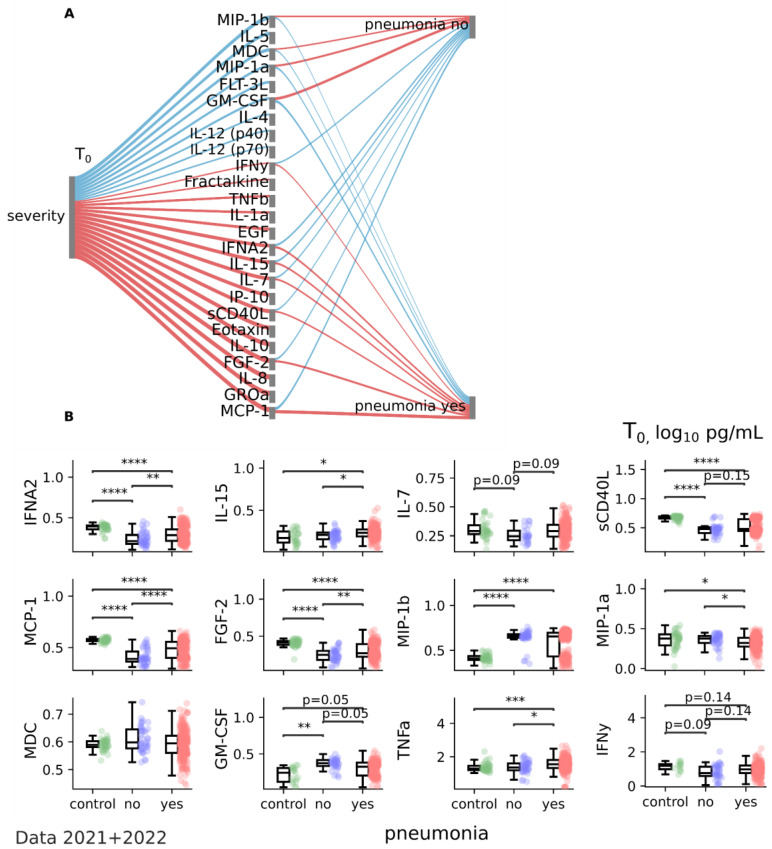
Correlation analysis of cytokine patterns of dependence on coronavirus infection with and without pneumonia for data collected in 2021 and 2022 at T0 (1–3 days after illness onset). (**A**) Sankey diagram depicting the significant associations between severity, cytokine profile, and pneumonia status. Positive associations are depicted in red and negative associations in blue. Positive association indicates that both variables increase and decrease together. Negative association indicates that while one variable is increasing the other is decreasing and vice versa. The width of the flow indicates the strength of the association (effect size). Spearmanr coefficients for continuous variables and Point-Biserial coefficients for binary-continuous pairs, with permutation (1000 repeats), FDR, *p* < 0.05. (**B**) Boxplots of cytokine values (log10 pg/mL) in control, pneumonia-no, and pneumonia-yes groups with indication of significance. Control data are shown in green, pneumonia-no data in blue, and pneumonia-yes data in red. Kruskal–Wallis with a post hoc Dunn’s test with FDR correction. * *p* < 0.05; ** *p* < 0.01; *** *p* < 0.0001; **** *p* < 0.00001. Data 2021 + 2022 (Supplementary Table S2).

Analysis of serum cytokines from patients with and without pneumonia also demonstrated a clear difference (Figure 3A). Coronavirus infection with pneumonia positively correlates with pro-inflammatory IFNA2 (*p* = 0.0051), IL-15 (*p* = 0.0087), IL-7 (*p* = 0.0333), sCD40L (*p* = 0.0336), MCP-1 (*p* = 0.0001), and anti-inflammatory FGF-2 (*p* = 0.0066) and negatively correlates with pro-inflammatory MIP-1b (*p* = 0.049), MIP-1a (*p* = 0.0093), MDC (*p* = 0.055), and GM-CSF (*p* = 0.0045) (Figure 3A,B, Table 2).

**Figure 4:**
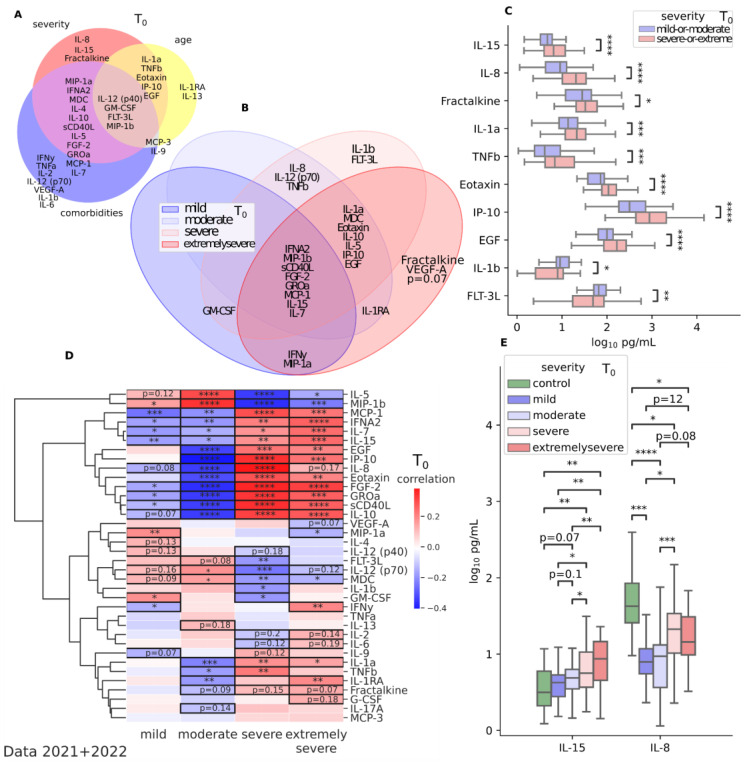
Significant cytokine shifts associated with age, disease severity, and presence of comorbid pathologies for data collected in 2021 and 2022 at T0 (1–3 days after illness onset). (**A**,**B**) Venn diagrams of significant cytokine shifts in serum showing overlapping and distinct shifts between subgroups based on (**A**) age, comorbidities, and severity and (**B**) different severity levels. (**C**) Boxplots of cytokine levels in two combined severity subgroups (mild/moderate vs. severe/extremely severe). Mann–Whitney-U test with FDR correction; (**D**) Correlation heatmap with dendrogram shows cytokines that are significantly associated with disease severity (relative to other groups). Positive associations are depicted in red and negative associations in blue. Positive association indicates that values in one group increase or decrease together relative to all other groups’ data. Negative association indicates that values in one group increase or decrease in opposite directions in relation to all other groups’ data. Spearmanr coefficients for continuous variables and Point-Biserial coefficients for binary-continuous pairs, FDR, *p* < 0.05; (**E**) Boxplots of cytokine levels in all severity groups compared to control for cytokines that were found to be associated with disease severity independent of age or comorbidities. Kruskal–Wallis with a Dunn’s post hoc multiple comparison test, FDR, *p* < 0.05. * *p* < 0.05; ** *p* < 0.01; *** *p* < 0.0001; **** *p* < 0.00001. Data 2021 + 2022.

We have identified cytokines that characterize acute coronavirus infection and are significant in terms of the severity of the disease, age, and the presence of comorbid conditions such as arterial hypertension, diabetes mellitus, overweight, and chronic heart failure. According to the data obtained, the severity of SARS-CoV-2 infection is determined by elevated values of the pro-inflammatory cytokines IL-15 (*p* < 0.0001), IL-8 (*p* = 0.0009), and fractalkine (*p* = 0.0535) independent of age and comorbid conditions (Figure 4A,E).

Cytokines associated with disease severity in older patients with more than one underlying pathology include FLT-3L (*p* = 0.0016), MIP-1b (*p* < 0.0001), GM-CSF (*p* = 0.0109), and IL-12(p40) (*p* = 0.026) (Figure 4A); in addition, in our study, the levels of these cytokines increased compared to healthy controls (Figure 2B). Figure 4B shows cytokines that are significantly associated with disease severity, and the Venn diagram illustrates the overlap of these associations across severity groups.

The severe and extremely severe forms of disease compared to other forms are characterized by a significant release of the following pro-inflammatory cytokines (Table 3): MCP-1 (*p* < 0.0001 and *p* = 0.0007), IFNa2 (*p* = 0.0016 and *p* < 0.0001), IL-7 (*p* = 0.0199 and *p* = 0.0008), IL-15 (*p* = 0.0073 and *p* = 0.0001), EGF (*p* = 0.0002 and *p* = 0.0017), IP-10 (*p* < 0.0001 and *p* < 0.0001), IL-8 (*p* < 0.0001 and *p* = 0.17), Eotaxin (*p* < 0.0001 and *p* = 0.0031), FGF-2 (*p* < 0.0001 and *p* < 0.0001), GROa (*p* < 0.0001 and *p* < 0.0001), sCD40L (*p* < 0.0001 and *p* = 0.0001), and IL-10 (*p* < 0.0001 and *p* < 0.0001). All of these cytokines are significantly reduced in mild to moderate forms of coronavirus infection (Figure 4C). While cytokine levels in moderate cases may not reach the levels seen in severe cases, they can still contribute to tissue damage and clinical symptoms. Regardless of the severity of the disease, the majority of patients experience shifts in IL-15, MCP-1, MIP-1b, IFNA2, FGF-2, sCD40L, IL-10, and GROa compared to the control (Figure 4B–D).

### 3.3. Cytokines Pattern of Post-COVID Syndrome or Post-Acute Sequelae of SARS-CoV-2 Infection (PASC)

**Figure 5:**
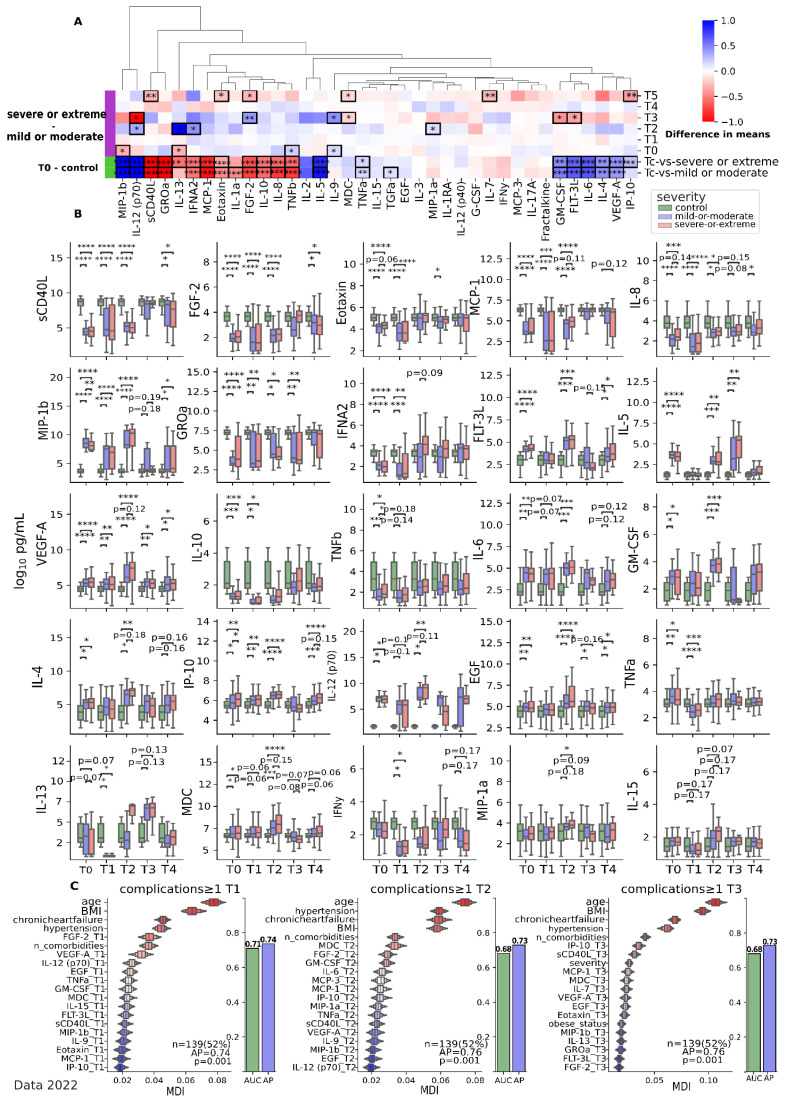
Dynamic variations in changes in cytokines at points T1-T5 for data collected in 2022. (**A**) Dynamic differences in means between severe/extreme and mild/moderate disease cytokine levels (log10 pg/mL). Mann–Whitney-U test with FDR correction (Supplementary Table S3). (**B**) Boxplots of cytokine levels (log10 pg/mL) with indication of significance in two combined severity subgroups (severe/extreme and mild/moderate) compared to the control in dynamics. Kruskal–Wallis with a Dunn’s *post hoc* multiple comparison test, FDR (Supplementary Table S4). (**C**) Ordination of features by importance (↑) for discriminating between two subgroups (complications ≥ 1) identified at T1, T2, and T3 using random forest analysis with leave-one-out cross-validation. Importance is inferred based on Mean Decrease of Impurity (MDI) (↑) values. * *p* < 0.05; ** *p* < 0.01; *** *p* < 0.0001; **** *p* < 0.00001. Data 2022. T1—15th day of observation (acute phase); T2—1 month of observation; T3—3 months of observation; T4—6 months of observation; T5—9 months of observation.

The immune system response post-COVID is different from the immune response seen in acute COVID-19. At the end of the acute phase (T1) of coronavirus infection (COVID-19), significant changes in cytokine levels were observed. Key cytokines (Figure 5C, T1) such as FGF-2 (*p* < 0.0001), VEGF-A (*p* = 0.11), EGF (*p* < 0.0001), and IL-12(p70) (*p* < 0.0001) showed noteworthy alterations (compared to T0). FGF-2 levels were reduced compared to the initial time point (T0) (pT1 < 0.0001), but there was a tendency towards a significant increase (Figure 5B) in subsequent observations (pT2 = 0.4, pT3 < 0.0001, pT4 < 0.0001, pT5 < 0.0001).

In subsequent time points, there were distinct differences in cytokine changes between patients with mild/moderate disease and those with severe/extremely severe disease (Figure 5A,B). During the T2 time point, several cytokines demonstrated significant variations between these groups (Table 3), including IL-13 (*p* = 0.0284), IFNa2 (*p* = 0.01), MIP-1a (*p* = 0.0279), IL-4 (*p* = 0.061), IL-12(p70) (*p* = 0.0181), MCP-1 (*p* = 0.0767), IL-1a (*p* = 0.0764), MDC (*p* = 0.12), IL-15 (*p* = 0.076), and VEGF-A (*p* = 0.076). These changes indicate ongoing immune responses and potential tissue regeneration processes. Notably, IL-6 (pmild/moderate = 0.003 and psevere/extreme = 0.0051), MDC (pmild/moderate = 0.0002 and psevere/extreme < 0.0001), and FGF-2 (pmild/moderate < 0.0001 and psevere/extreme < 0.0001) levels were elevated during this phase compared to the control, particularly in patients with severe/extremely severe disease. Elevated levels of IL-6 and MDC suggest persistent inflammation and ongoing activation of immune cells to clear viral remnants. Increased FGF-2 levels may indicate the activation of tissue repair mechanisms (Figure 5A,B). At the T3 time point, a decrease in IP-10 (pmild/moderate < 0.0001 and psevere/extreme < 0.0001) levels compared to the acute phase was observed, primarily in patients with severe/extremely severe disease. This reduction suggests a decline in inflammatory activity. Conversely, there was a significant increase in sCD40L levels (pmild/moderate < 0.0001 and psevere/extreme = 0.0003), indicating an ongoing active inflammatory process. Interestingly, the elevation of sCD40L (*p* = 0.2) was not dependent on the severity of the infection (Figure 5B).

**Figure 6:**
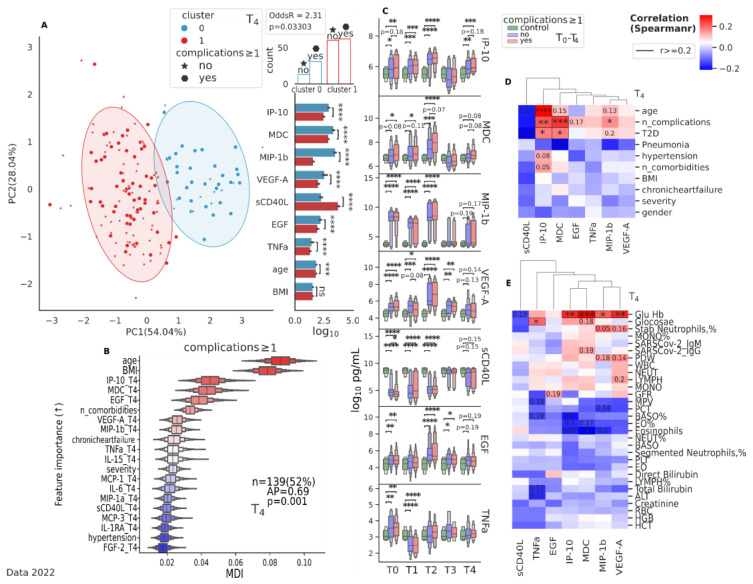
Differentiation between subgroups (complications ≥ 1) at T4 (6 months of observation) for data collected in 2022. (**A**) Principal component analysis (PCA) projection with clustering based on the three major cytokines identified as important for differentiating between subgroups (complications ≥ 1) at T4 (IP-10 + MDC + EGF). A PCA was performed on log10 transformed standard scaled data. Subsequently, agglomerative clustering (k = 2) with “ward” linkage was used to identify clusters. For count analysis, a Fisher’s exact test was used, and for group analysis, a Mann–Whitney U test was used with FDR correction *p* ≤ 0.05. (**B**) Ordination of features by importance for differentiating between subgroups (complications ≥ 1) at T4 using a random forest analysis with leave-one-out cross-validation, with a permutation test (with 1000 repeats and five stratified splits) for the most important features identified. (**C**) Boxplot of cytokine levels (log10 pg/mL) for key parameters over T0-T4 compared to the control. Kruskal–Wallis with a Dunn’s *post hoc* multiple comparisons test, FDR *p* ≤ 0.5. Correlation heatmap (**D**) between demographic parameters and cytokine levels at T4 and (**E**) between clinical parameters and cytokine levels at T4. Positive associations are depicted in red and negative associations in blue. Positive association indicates that both variables increase and decrease together. Negative association indicates that while one variable is increasing the other is decreasing and vice versa. Spearmanr coefficient for continuous variables and Point-Biserial coefficient for binary-continuous pairs, FDR, *p* < 0.05. Data 2022. * *p* < 0.05; ** *p* < 0.01; *** *p* < 0.0001; **** *p* < 0.00001. Data 2021 + 2022 (Supplementary Table S5).

Regardless of the severity of the coronavirus infection, by 6 months of observation (T4), we established a clear clustering of changes in the cytokine pattern (Figure 6), where EGF (*p* < 0.0001), MDC (*p* < 0.0001), TNFa (*p* < 0.0001), IP-10 (*p* < 0.0001), sCD40L (*p* < 0.0001), and VEGF-A (*p* < 0.0001) were found to be distributed significantly differently between the clusters (Figure 5B,C and Figure 6A). These cytokines in the study group are higher at the T4 point compared to the control, with the exception of sCD40L. Further analysis of cytokine levels in groups subdivided by the presence of complications at T4 showed little or no significant results (Figure 6C).

**Figure 7:**
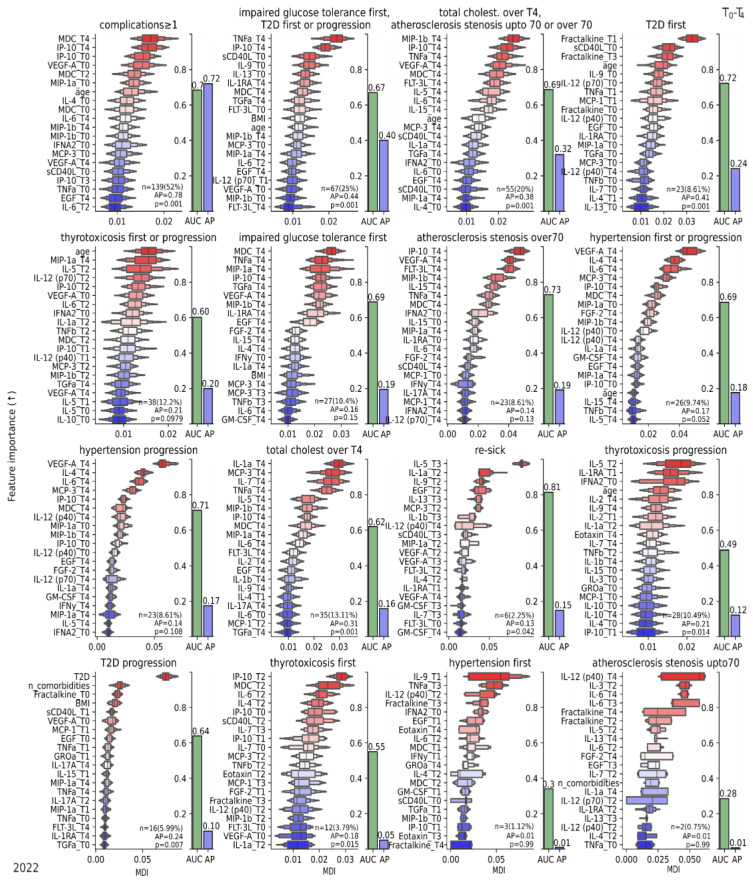
Ordination of features by importance for discriminating between subgroups (complications ≥ 1) and specific complications (indicated in the title) at T4 using all data from T0–T4. Permutation importance and class balance are shown in the figure (bottom right). Bars indicate the score obtained in leave-one-out cross-validation. Importance is inferred based on Mean Decrease of Impurity (MDI) (↑) values using a random forest analysis with leave-one-out cross-validation. Data 2022. T1—15th day of observation (acute phase); T2—1 month of observation; T3—3 months of observation; T4—6 months of observation.

Moreover, at the T4 time point, several complications were diagnosed in the patients under observation. These complications included arterial hypertension, type 2 diabetes mellitus, glucose tolerance, thyrotoxicosis, atherosclerosis, and rapid progression of previously diagnosed pathologies. Specific combinations of cytokines were associated with certain conditions (Figure 7). The combination of IP-10, FLT-3L, VEGF-A, and MIP-1b was characteristic of atherosclerosis with stenosis above 70. These cytokines contribute to immune cell recruitment, angiogenesis, and inflammation, which are associated with plaque growth and instability.

On the other hand, IL-12 (p70), fractalkine, IL-6, and IL-3 are associated with atherosclerosis with stenosis below 70%, where IL-6 can promote endothelial dysfunction and fractalkine can contribute to the early stages of plaque development and mild stenosis. In the case of patients experiencing both a combined increase in total cholesterol and atherosclerosis with stenosis above 70, elevated levels of the cytokines MIP-1b, TNFa, IP-10, and VEGF-A were observed at the T4 time point.

Hypertension progression was associated with VEGF-A, IL-6, IL-4, and MCP-3, while newly detected hypertension was linked to IL-9, TNFa, IL12(p40), IFNa2, and EGF. Hypertension involves chronic low-grade inflammation, and these factors may contribute to vascular remodeling, endothelial dysfunction, and increased blood pressure. Complications in diabetes mellitus were correlated with elevated levels of fractalkine at T1. Furthermore, shifts in TNFa and IP-10 at T4 and sCD40L, IL-9, and IL-13 at T0 were associated with diabetes mellitus progression with impaired glucose tolerance.

Elevated levels of IFNa2 at T0 and IL-5 at T2 were associated with the progression of thyrotoxicosis. Additionally, significant cytokines related to newly detected thyrotoxicosis were observed at the T2 time point, including IP-10, MDC, IL-6, and IL-4. The presence of these cytokines suggests an ongoing immune activation and inflammatory state associated with the onset of thyrotoxicosis.

During the observation period T2–T4, some patients experienced re-infection with SARS-CoV-2, and their cytokine profiles showed significant changes. The levels of IL-5, IL-1a, IL-9, and EGF at T2 and IL-1b and IL-13 at T3 were found to be significantly altered in these patients. Re-infection with SARS-CoV-2 leads to distinct changes in the cytokine profile compared to the initial infection. These changes can be explained by several factors. First, immunological memory plays a significant role, as the immune system generates a more targeted response upon secondary exposure to the virus. This memory response can lead to a change in cytokine patterns compared to the primary infection. Second, the involvement of different viral strains may contribute to the observed variation. In this study, we have already demonstrated strain-specific immune responses, indicating that the body responds differently to different strains of SARS-CoV-2. Therefore, re-infection with a different strain can elicit unique cytokine responses.

In addition, the weakening of the immune system after the follow-up period of a few months as a result of sustained inflammation may also have contributed to the observed changes in the cytokine profile. These factors collectively explain the discrepancies observed in the cytokine profile upon re-infection with SARS-CoV-2 compared with primary infection. Further research is needed to fully understand the underlying mechanisms and their clinical implications.

**Figure 8:**
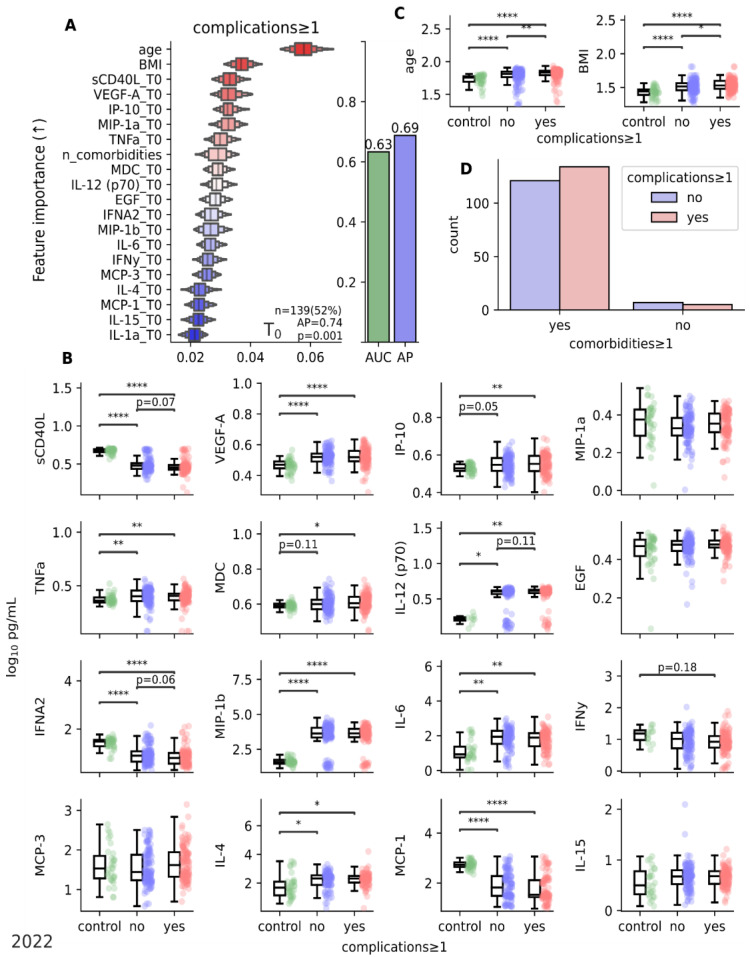
Analysis of predictive power of parameters at T0 for early prognosis of post-COVID complications (identified at T4). (**A**) Ordination of features by importance for detecting potential development of complications at T4 using early data at T0. Importance is inferred based on Mean Decrease of Impurity (MDI) (↑) values using a random forest analysis with leave-one-out cross-validation. (**B**) Boxplots of cytokine levels with indication of significance for the most important features at T0 in two subgroups (complications ≥ 1) compared to control. (**C**) Boxplots of age and BMI with indication of significance. Kruskal–Wallis with a Dunn’s *post hoc* multiple comparison test, FDR, *p* ≤ 0.5. (**D**) Count plot of association of occurrence between complications and comorbidities (including excess weight). * *p* < 0.05; ** *p* < 0.01; **** *p* < 0.00001. Data 2022. T1—15th day of observation (acute phase); T4—6 months of observation.

The obtained results were intended to develop a predictive framework for estimating the probability of post-COVID complications six months after the initial phase of contracting coronavirus (Figure 8A).

The predictive model indicated that older age and being overweight could influence the likelihood of experiencing post-COVID complications. Furthermore, based on the findings, it is crucial for clinicians to observe changes in cytokine profiles, specifically sCD40L, VEGF-A, IP-10, MIP-1a, and TNFa (Figure 8), during the acute phase of coronavirus infection.

## 4. Discussion

These findings highlight the complex and dynamic nature of cytokine responses in acute COVID-19, in the post-COVID period, and in re-infection scenarios, and their associations with disease severity, complications, and underlying health conditions.

It is crucial to highlight that some individuals may experience persistent symptoms and complications beyond the acute period; this phenomenon is often referred to as post-acute sequelae of SARS-CoV-2 infection (PASC). PASC immune response alterations, particularly the dysregulation of key cytokines such as FGF-2, VEGF-A, EGF, IL-12 (p70), IL-13, and IL-6, may play a critical role in the development of prolonged symptoms and chronic conditions [21,22].

Cytokine levels can vary greatly among individuals with COVID-19, and the cytokine response depends on the strain of the virus. Determined cytokines were associated with the severity of the disease, regardless of other factors such as age and comorbid conditions. Cytokine IL-15 is a critical innate immune regulatory cytokine with antiviral properties [23]. A number of authors have found that IL-15 is important as an early biomarker predicting progression to symptomatic and severe diseases, and is the best early predictor of the need for invasive ventilation [24,25]. Cytokine IL-8 is a pro-inflammatory chemokine associated with stimulation of neutrophil chemotaxis and degranulation, and has also been associated with disease severity in a number of studies [26,27]. The level of IL-8 rises before C-reactive protein. Fractalkine, also known as CX3CL1, is produced by alveolar macrophages and promotes the recruitment of monocytes and macrophages to the lungs [28]. Monserrat, J. et al. in their study showed that severe forms of COVID-19 are associated with reduced levels of fractalkine [29]. However, most other studies have shown that fractalkine levels were significantly elevated in patients with severe COVID-19 and may contribute to neurological vascular damage and thrombosis during SARS-CoV-2 infection [30,31,32]. Moreover, it has been shown that overexpression of fractalkine may promote the creation of a prothrombotic environment and enhance immune cell recruitment, leading to more severe COVID-19 and mortality [28]. Thus, fractalkine can serve as a marker of the severity of coronavirus infection and a prognostic marker for the development of thrombotic complications. Along with fractalkine, reduced VEGF-A is specific for the extremely severe form and IL-1b for the severe form. VEGF is primarily known for its role in the cardiovascular system, it also plays a role in the immune response to infection [32]. Although most studies reporting elevated levels of VEGF in severe cases of COVID-19, there are studies showing reduced VEGF levels in severe cases of COVID-19. Reduced expression of VEGF-A contributes to thrombosis by disrupting the normal function of the endothelium (the inner lining of blood vessels) [33]. Additionally, most studies show elevated levels of IL-1b, but in a retrospective study analyzing data from 39,539 COVID-19 patients, the levels of Il-1b were found to remain within the normal range [34]. Similar data were obtained for patients with coronavirus infection with coexisting arterial hypertension, diabetes mellitus, and hematological malignancies [35]. Pneumonia correlates with specific cytokines IFN2, Il-15, IL-7, sCD40L, MCP-1, FGF-2, MIP-1b, MIP-1a, MDC, and GM-CSF. The cytokine pattern also varies based on the presence of comorbidities and age.

The influence of age on the clinical course and prognosis of COVID-19 is a crucial consideration in our investigations. Immunosenescence, the natural age-related decline in immune function, may impact cytokine responses and disease severity during viral infections [36]. Advanced age has been associated with an elevated risk of severe disease progression and complications in COVID-19 patients [37,38].

The PASC immune response differs from acute infection, with alterations in key cytokines such as FGF-2, VEGF-A, EGF, IL-12(p70), IL-13, and IL-6.

A positive correlation between MDC and glycosylated hemoglobin levels was observed, as well as glucose levels. Increased MDC levels might indicate an ongoing inflammatory process, which can contribute to insulin resistance and subsequently elevated glucose levels. Furthermore, a negative correlation between MDC and eosinophil levels suggests that MDC is primarily involved in attracting macrophages and other immune cells to the site of inflammation rather than eosinophils. In turn, eosinophil is negatively correlated with IP-10.

Furthermore, TNF-alpha shows a positive correlation with glucose levels; it is known TNF-alpha has been shown to induce insulin resistance and impair glucose uptake in cells, leading to hyperglycemia. The negative correlations between TNF-alpha and total bilirubin, ALT, and MVP might be indicative of liver dysfunction (Figure 6E). It was previously shown that with SARS-CoV-2, moderately pronounced microvesicular deposits were determined in the liver tissue, which indicates liver damage. Damage to liver cells can occur as a result of various factors; it is believed that the virus can directly infect liver cells or cause an immune-mediated response that leads to inflammation and damage to the liver [39].

The presence of complications such as arterial hypertension, diabetes mellitus, and atherosclerosis is associated with distinct cytokine profiles. More interesting is the role of IL-13 in the development and progression of atherosclerosis. IL-13 exhibits a dichotomous role, initially linked to CD36 expression and foam cell formation [40,41]. However, further research uncovered its ability to maintain metabolic homeostasis by inducing PPARδ or PPARβ expression in adipose tissue, potentially promoting atherosclerosis [41,42].

Therefore, utilizing the obtained findings, we endeavored to construct a prognostic framework that enables us to anticipate the likelihood of post-COVID complications six months after the acute phase of coronavirus infection. Our predictive model (Figure 8A) supports that advanced age and being overweight serve as indicators that should raise concerns among clinicians regarding the potential for a severe progression and subsequent complications. It is important in the acute period of coronavirus infection to pay attention to changes in the structure of cytokines, including sCD40L, VEGF-A, IP-10, MIP-1a, and TNFa, in the presence of other factors such as age and comorbidity (Figure 8). The predictive model needs to be assessed and validated in independent studies. This is a promising task for future research.

There are several limitations. Firstly, the study was conducted in a single city and did not consider other factors such as geography or socio-economic determinants. As a result, the findings may not be representative of the broader population. Secondly, the study did not examine the dynamics of patients who contracted SARS-CoV-2 infection in 2021. Since cytokine profiles can differ between those who recovered in 2021 and 2022, the progression and complications may also vary. Therefore, the findings of this study may not accurately reflect the current situation. Thirdly, the study specifically focuses on a six-month time frame after the acute phase of COVID-19 infection. It is important to acknowledge that the long-term effects and complications of COVID-19 can extend beyond this period, and the study does not account for these potential outcomes.

## 5. Conclusions

This study revealed significant differences in cytokine profiles between COVID-19 patients and the control group, as well as variations between patients infected with different strains and those with different severities of the disease. Cytokines played a crucial role in determining age-related complications, the development of PASC, and the severity of COVID-19. These findings contribute to a better understanding of the immune response and potential prognostic markers in COVID-19 patients.

## Figures and Tables

**Table 1:** Baseline demographics and clinical characteristics.

Parameter M ± Sd (IQR)/Counts	Control (*n* = 40)	Mild (*n* = 38)	Moderate (*n* = 165)	Severe (*n* = 71)	Extremely Severe (*n* = 20)	*p*-Value
Age (IQR) ^1^	53.0 ± 8.4 (48.2–59.0)	51.6 ± 16.1 (39.5–65.0)	54.1 ± 15.0 (45.0–66.0)	61.7 ± 13.2 (56.5–70.0)	65.3 ± 14.1 (58.0–73.0)	**0.007 ^a^**
Gender, M/F (M%) ^2^	21/19 (52.5%)	16/22 (42.1%)	64/101 (38.9%)	22/49 (31.5%)	13/7 (65%)	**0.036 ^b^**
BMI (IQR) ^1^	26.3 ± 4.4 (23.6–28.6)	27.4 ± 5.4 (24.0–30.2)	28.8 ± 6.8 (24.2–32.4)	29.1 ± 6.1 (24.8–32.4)	30.2 ± 5.2 (25.9–33.4)	**0.028 ^a^**
Year of observation 2021/2022 (2021%) ^2^	-	0/38 (0%)	0/165 (0%)	35/36 (49.29%)	15/5 (75%)	**≤0.0001 ^b^, 0.046 ^c^**
Pneumonia (Yes%) ^2^	-	4/34 (10.5%)	162/3 (98.2%)	68/3 (95.7%)	20/0 (100%)	**≤0.0001 ^b^**
PCR+/− (Positive%) ^2^	-	23/15 (60.5%)	121/44 (73.3%)	23/48 (32%)	5/15 (25%)	**≤0.0001 ^b^**
Normal/Overweight/Obesity (Overweight or Obese%) ^2^	-	13/10/15 (65.7%)	48/66/51 (70.1%)	20/27/24 (71.8%)	5/10/5 (75%)	**0.007 ^b^**
Hyper-/Normotension (Yes%) ^2^	-	21/17 (55.3%)	85/80 (51.5%)	20/51 (28.2%)	5/15 (25%)	**0.001 ^b^**
Type II diabetes Yes/No (Yes%) ^2^	-	3/35 (7.9%)	22/143 (13.3%)	12/59 (16.9%)	4/16 (20%)	0.51 ^b^
Chronic heart failure (Yes%) ^2^	-	5/33 (13.2%)	22/143 (13.3%)	15/56 (21.2%)	3/17 (15%)	0.48 ^b^
Comorbidities ≥ 1 (Yes%) ^2^	-	35/3 (92.1%)	143/22 (86.7%)	61/10 (85.9%)	17/3 (85%)	0.79 ^b^

IQR = Interquartile Range; BMI = Body Mass Index; ^1^ Mean ± Sd (IQR); ^2^ Counts (Percentage %); ^a^ Determined by the Kruskal–Wallis test; ^b^ Determined by the Chi-squared test; ^c^ Determined by the Fisher exact test for Severe vs. Extremely Severe pair; Age: Control vs. Severe: *p* ≤ 0.01, Mild vs. Severe: *p* ≤ 0.01, Moderate vs. Severe: *p* ≤ 0.01, Control vs. Severe: *p* ≤ 0.01, Mild vs. Extremely Severe: *p* ≤ 0.01, Moderate vs. Extremely Severe: *p* ≤ 0.01, else: NS by Dunn’s *post hoc* multiple comparison test; BMI: Control vs. Severe: *p* ≤ 0.05, Control vs. Extremely Severe: *p* ≤ 0.05, Mild vs. Extremely Severe: *p* ≤ 0.05, else: NS by Dunn’s *post hoc* multiple comparison test.

**Table 2:** Comparison of cytokine levels in subgroups.

Control vs. T0 ^a^			Control vs. T0 ^a^		Pneumonia-No vs. Pneumonia-Yes in T0 ^b^	
	*p*-Value_2021_	*p*-Value_2022_		*p*-Value		*p*-Value
pro-inflammatory, 2021 + 2022 (↑)			2021 (↑)–2022 (↓), 2022 (↓):		pro-inflammatory, pneumonia-yes (↑)	
TNFa	0.034	0.0007	FGF-2	<0.0001	IFNA2	0.0051
IL-6	0.14	0.0009	IFNA2	<0.0001	IL-15	0.0087
IL-1RA	0.0051	0.73	IL-1a	<0.0001	IL-7	0.0333
IL-15	<0.0001	0.1	IL-7	<0.0001	sCD40L	0.0336
IL-2	0.5	0.4	IL-10	<0.0001	MCP-1	0.0001
IL-5	0.51	<0.0001	2021 (↓)–2022 (↑), 2021 (↓):		anti-inflammatory, pneumonia-yes (↑)	
MIP-1b	0.85	<0.0001	IL-1b	0.0548	FGF-2	0.0066
anti-inflammatory, 2021 + 2022 (↑)			IL-12	0.0001	pro-inflammatory, pneumonia-yes (↓)	
IL-4	0.25	0.0033	MDC	<0.0001	MIP-1b	0.049
IP-10	<0.0001	0.0051	2021 (=)–2022 (↓), 2022 (↓):		MIP-1a	0.0093
TGFa	0.0002	0.069	Fractalkin	0.0023	MDC	0.055
EGF	<0.0001	0.45	GM-CSF	<0.0001	GM-CSF	0.0045
VEGF-A	0.0023	0.0001	GROa	<0.0001		
2021 + 2022 (↓)			IL-12 (p70)	<0.0001		
MIP-1a	<0.0001	0.22				
IL-8	0.13	<0.0001				
IL-9	0.29	0.74				
MCP-1	0.75	0.0001				
Eotaxin	0.33	<0.0001				
IFNgamma	0.53	0.14				
sCD40L	0.4	<0.0001				
TNFb	0.82	0.0036				

(↑)—level increased compared to control; (↓)—level decreased compared to control; (=)—same level as control; Pneumonia-yes (↑)—level increased in patients with pneumonia; Pneumonia-yes (↓)—level decreased in patients with pneumonia; ^a^ Determined by a Kruskal–Wallis test with Dunn’s *post hoc* multiple comparison test with FDR adjustment; ^b^ Determined by a Point-Biserial test on log10 transformed data with FDR adjustment.

**Table 3:** Comparison of cytokine levels in subgroups.

Severe or Extremely Severe vs. Mild/Moderate in T0 ^a^			Severe/Extremely Severe in T0 vs. Severe/Extremely Severe in T1 ^b^		Mild/Moderate in T2 vs. Severe/Extremely Severe in T2 ^b^	
	*p*-Value (Severe)	*p*-Value (Extremely Severe)		*p*-Value		*p*-Value
Pro-Inflammatory (↑)			(↓)		Severe/Extremely Severe (↑)	
MCP-1	<0.0001	0.0007	FGF-2	<0.0001	IL-13	0.0284
IFNa2	0.0016	0.0001	VEGF-A	0.11	IFNa2	0.01
IL-7	0.0199	0.0008	EGF	<0.0001	MIP-1a	0.0279
IL-15	0.0073	0.0001	IL-12(p70)	<0.0001	IL-4	0.061
EGF	0.0002	0.0017			IL-12(p70)	0.0181
IP-10	<0.0001	<0.0001			MCP-1	0.0767
IL-8	<0.0001	0.17			IL-1a	0.0764
Eotaxin	<0.0001	0.0031			MDC	0.12
FGF-2	<0.0001	<0.0001			IL-15	0.076
GROa	<0.0001	<0.0001			VEGF-A	0.076
sCD40L	<0.0001	0.0001				
IL-10	<0.0001	<0.0001				

(↑)—level increased; (↓)—level decreased; ^a^ determined by a Kruskal–Wallis test with a Dunn’s *post hoc* multiple comparison test with FDR adjustment; ^b^ determined by a Mann–Whitney test with FDR adjustment.

## Data Availability

All data that underlie the results reported in this manuscript will be available to the scientific community on request to the corresponding authors. Data will be available indefinitely after the publication of the manuscript.

## Supplementary Materials

The following supporting information can be downloaded at: https://www.mdpi.com/article/10.3390/jcm12165224/s1, Table S1: Cytokine profile by year of observation; Table S2: Correlation analysis of cytokine patterns of dependence on coronavirus infection with and without pneumonia; Table S3: Dynamic variations in changes in cytokines at points T1–T5; Table S4: Kruskal–Wallis with a Dunn’s *post hoc* multiple comparison test; Table S5: Differentiation between subgroups (complications ≥ 1) at T4.
